# Efficacy and safety of Danhong injection on inflammatory factors and vascular endothelial function in patients with unstable angina pectoris: a systematic review and meta-analysis of randomized clinical trials

**DOI:** 10.3389/fphar.2025.1389746

**Published:** 2025-06-13

**Authors:** Zunqi Kan, Wenli Yan, Cong Chen, Huanyu Gao, Yongmei Song

**Affiliations:** ^1^ Guang’anmen Hospital, China Academy of Chinese Medical Sciences, Beijing, China; ^2^ School of Acupuncture-Moxibustion and Tuina, International Acupuncture and Moxibustion Innovation Institute, Beijing University of Chinese Medicine, Beijing, China; ^3^ Institute for Literature and Culture of Chinese Medicine, Shandong University of Traditional Chinese Medicine, Jinan, Shandong, China

**Keywords:** Danhong injection, unstable angina pectoris, inflammatory factors, vascular endothelial function, meta-analysis

## Abstract

**Background:**

Inflammatory factors and vascular endothelial damage are significantly involved in the development of unstable angina pectoris (UAP). Danhong injection (DHI) is a compound injection composed of *Salvia miltiorrhiza* and *Carthamus tinctorius* extracts. Several clinical studies have demonstrated DHI’s efficacy for treating UAP, with potential pharmacological effects on inflammatory factors and vascular endothelial function. However, to date, the current evidence has not been systematically summarized and analyzed.

**Purpose:**

This study provides a comprehensive overview of the most recent clinical findings and systematically analyzes the impact of DHI on inflammatory factors and vascular endothelial function among individuals diagnosed with UAP.

**Methods:**

We searched for randomized controlled trials (RCTs) conducted until January 2023 from two clinical trial registries and eight literature databases. The Cochrane Risk of Bias Tool 2.0 was utilized to assess the potential bias in the included trials, while the GRADE system was employed to evaluate the outcome quality.

**Results:**

We included 46 trials involving 4,601 patients with UAP. The meta-analysis results suggested that DHI significantly reduced the levels of high-sensitivity C-reactive protein (hs-CRP) (standard mean difference [SMD] = 1.34, 95% confidence interval [CI] [-1.77, −0.90], P < 0.00001), tumor necrosis factor-alpha (TNF-α) (SMD = −0.84, 95% CI [-1.54, −0.15], P = 0.02), interleukin-6 (IL-6) (SMD = −1.05, 95% CI [-1.86, −0.25], P = 0.01), endothelin/endothelin-1 (ET/ET-1) (SMD = −2.01, 95% CI [-2.57, −1.46], P < 0.00001), and homocysteine (Hcy) (SMD = −0.55, 95% CI [-0.71, −0.39], P < 0.00001) but increased the nitric oxide (NO) level (SMD = 1.51, 95% CI [1.04, 1.97], P < 0.00001) in patients with UAP. Twenty-one RCTs described adverse events.

**Conclusion:**

DHI effectively and safely reduced hs-CRP, TNF-α, IL-6, ET/ET-1, and Hcy levels and increased the NO level in patients with UAP. However, considering the overall low quality of the original studies, future large-scale, high-quality RCTs are imperative to provide robust evidence for clinical practice.

**Systematic Review Registration:**

identifier CRD42023391497.

## 1 Introduction

Coronary artery disease (CAD) is a significant contributor to global morbidity and mortality (Duan et al., 2018). Unstable angina pectoris (UAP) is an acute coronary syndrome in CAD and a clinical symptom between stable angina pectoris and acute myocardial infarction. Although the UAP-related mortality rate has declined annually in recent years, the prevalence rate remains high; therefore, it remains a major public health concern (Man et al., 2020; Li M. et al., 2022). UAP should be suspected if a patient presents with resting angina for >20 min, crescendo angina, or new-onset angina (Collet et al., 2021). UAP mainly lasts for a long time at rest or at night, and because of its special physiological mechanisms and clinical manifestations, it can develop into acute myocardial infarction or sudden death if not treated in time (Guo J. et al., 2020). The pathology of UAP is thought to be related to local coronary atherosclerotic lesions (Kosiński et al., 2022), such as vasospasm, intravascular inflammation, thrombosis, and unstable plaques. These conditions can lead to vascular stenosis or blockage, resulting in myocardial ischemia. Inflammation significantly affects cardiovascular diseases; recently, addressing inflammation has become a potential way to prevent such diseases (Wolf and Ley, 2019). Evidence suggests that inflammation is caused by substances responsible for vascular plaque formation. Furthermore, inflammatory mediators and cytokines induce endothelial dysfunction and platelet adhesion, ultimately leading to the progression of atherosclerosis (Bi et al., 2019). The combined effect of inhibiting inflammation and oxidative stress, as well as improving endothelial function, can provide a favorable microenvironment for endothelial injury repair and potentially alleviate atherosclerotic lesions. Conventional Western medical treatments, such as plaque stabilization and antiplatelet and antithrombotic drugs, have been successfully used to reduce angina. However, owing to the complexity of UAP, the long-term use of these drugs can produce side effects and resistance that influence the treatment’s effectiveness (Duan et al., 2018; Xi et al., 2023). Therefore, it is crucial to investigate and identify other novel and effective treatments for UAP.

Danhong injection (DHI) is a standardized traditional Chinese medicine product. According to the traditional Chinese medicine theory, the pathogenesis of UAP is closely associated with blood coagulation, and the main function of DHI is to promote blood circulation, blood stasis, and dredging meridians (Wu et al., 2015); thus, it is effective against UAP (Wu et al., 2017; Guo S. et al., 2020; Xing et al., 2021). DHI is a proprietary Chinese medicine extracted from *Salvia miltiorrhiza* Bunge (family: Lamiaceae; red sage or Danshen in Chinese) and *Carthamus tinctorius* L. (family: Compositae; safflower or Honghua in Chinese) (Zou et al., 2018). The main bioactive ingredients include salvianolic acids, danshensu, protocatechuic aldehyde, rosmarinic acid, caffeic acid, and hydroxysafflor yellow A (Feng et al., 2019). Contemporary pharmacological investigations have revealed that DHI and its active components elicit pleiotropic protective effects in the cardiovascular system, such as anti-fibrosis, reducing blood viscosity, anti-inflammation, anti-oxidative damage, improving endothelial function and alleviate endothelial dysfunction (Lyu et al., 2017; Yi et al., 2018; Hu et al., 2019; Nijat et al., 2022).

Recent studies have found that DHI is effective for patients with cardiovascular and cerebrovascular diseases, such as angina pectoris, myocardial infarction, and cerebral infarction (Yu et al., 2018; Liu et al., 2022). However, a comprehensive systematic assessment of the effects of DHI on inflammatory factors and endothelial function in patients with UAP is lacking. Therefore, we performed a comprehensive systematic review and meta-analysis to thoroughly assess the impact of DHI on inflammatory factors and vascular endothelial function in patients with UAP, aiming to offer robust clinical evidence for DHI.

## 2 Methods

The meta-analysis was facilitated by the prospective registration of the review protocol with PROSPERO (No: CRD42023391497). The study was carried out in accordance with the Cochrane Handbook for Systematic Reviews of Interventions and documented following the guidelines provided by the Preferred Reporting Items for Systematic Reviews and Meta-Analyses (PRISMA) (Page et al., 2021).

### 2.1 Data sources and search strategy

The following databases were comprehensively searched from their inception until January 15, 2023: PubMed, Embase, Web of Science, Cochrane Library, China National Knowledge Infrastructure, Wanfang Data, China Biomedical Literature Database, and Chongqing VIP Information. The Chinese Clinical Trial Registry and ClinicalTrials.gov were also comprehensively searched. There were no constraints on publication date, language, or publication status of the included studies. The main search terms used were “Danhong injection” and “unstable angina pectoris.” Supplementary Table S1 presents the detailed search strategies for all databases.

### 2.2 Eligibility criteria for included studies

The RCTs included in this study fulfilled the PICOS criteria.

#### 2.2.1 Inclusion criteria

Studies were included based on the following Population, Intervention, Comparison, Outcomes, and Study (i.e., PICOS) criteria:

1) Type of participant (P): Patients receiving DHI. All patients, regardless of age, sex, or ethnicity, who fulfilled at least one of the guidelines for DHI established by the European Society of Cardiology (Messerli et al., 2006), World Health Organization, Chinese Society of Cardiology (Ke and Chen, 2007), or American College of Cardiology Foundation/American Heart Association (Braunwald et al., 2002).
2) Types of intervention (I): DHI alone or combined with conventional therapy.
3) Types of comparators (C): Conventional Western medicine (CWM), including aspirin, β-blockers, statins, nitrates, calcium channel blockers, angiotensin-converting enzyme inhibitors, angiotensin receptor blockers, or low-molecular-weight heparin.
4) Types of outcome measures (O): The primary outcomes focused on (1) inflammatory factors, including high-sensitivity C-reactive protein (hs-CRP), tumor necrosis factor-α (TNF-α), and interleukin-6 (IL-6) and (2) indicators of vascular endothelial function, such as nitric oxide (NO), endothelin/endothelin-1 (ET/ET-1), and homocysteine (Hcy). The secondary outcomes were factors related to adverse events (AEs).
5) Types of studies (S): Randomized controlled trials (RCTs) with unrestricted language and methods.

#### 2.2.2 Exclusion criteria

The following study types were excluded: (1) non-RCTs, animal experimental studies, case reports, protocols, reviews, and conference abstracts; (2) duplicate publications and those with no full text, incomplete or incorrect data, and extraneous interventions; and (3) those with no relevant outcome measures.

### 2.3 Data extraction

Based on the aforementioned standards, data were independently extracted by two reviewers. The extracted data included language, the first author, publication year, baseline characteristics, details of the interventions, and outcome measurements. The results underwent cross-validation at every stage of the procedure, and any inconsistencies were resolved through discussion and agreement with an arbiter.

### 2.4 Risk of bias assessment

The Cochrane Risk of Bias Tool 2.0 (Sterne et al., 2019) was utilized to assess the potential for bias in the included trials. The assessment criteria encompassed the randomization process, deviations from the intended interventions, missing outcome data, measurement of outcome, and selection of the reported result. The domains were classified into three categories based on their risk level: “low risk,” “some concerns,” or “high risk.” Any discrepancies in the evaluation were resolved through deliberation and agreement with a mediator.

### 2.5 Data analysis

In this meta-analysis, STATA 17.0 (Higgins et al., 2011) and Review Manager software 5.4 were used for the statistical analyses of primary and secondary outcomes. Dichotomous variables were evaluated using risk ratios (RR), whereas continuous variables were examined by calculating the mean difference or standard mean difference (SMD). The heterogeneity among the studies was evaluated using the Q test and I^2^ statistics. Depending on the heterogeneity test result, a random or fixed-effects model was selected for the data analysis; the fixed-effects model was used if no significant heterogeneity was detected (P ≥ 0.10 or I^2^ ≤ 50%). Conversely, the random-effects model was employed in cases of observed heterogeneity. The results were determined using P-values, with statistical significance defined as P < 0.05. Additionally, the total DHI dose, detection methods, and detection time were considered potential influential factors based on the included studies. A meta-regression analysis was performed to investigate potential factors contributing to the observed heterogeneity. The relevant factors identified in the meta-regression analysis were used as classification criteria for the subgroup analysis. To assess the reliability of the merged results, a sensitivity analysis was performed where each study was removed one by one. Furthermore, publication bias was identified by employing funnel plots, an Egger’s test, and a Begger’s test.

### 2.6 Certainty assessment

In this study, the certainty of evidence was evaluated using the grading of recommendations, assessment, development, and evaluation (GRADE) system (Murad et al., 2017). The level of certainty in the evidence was rated as “high,” “medium,” “low,” or “very low.”

## 3 Results

### 3.1 Study selection

**FIGURE 1:**
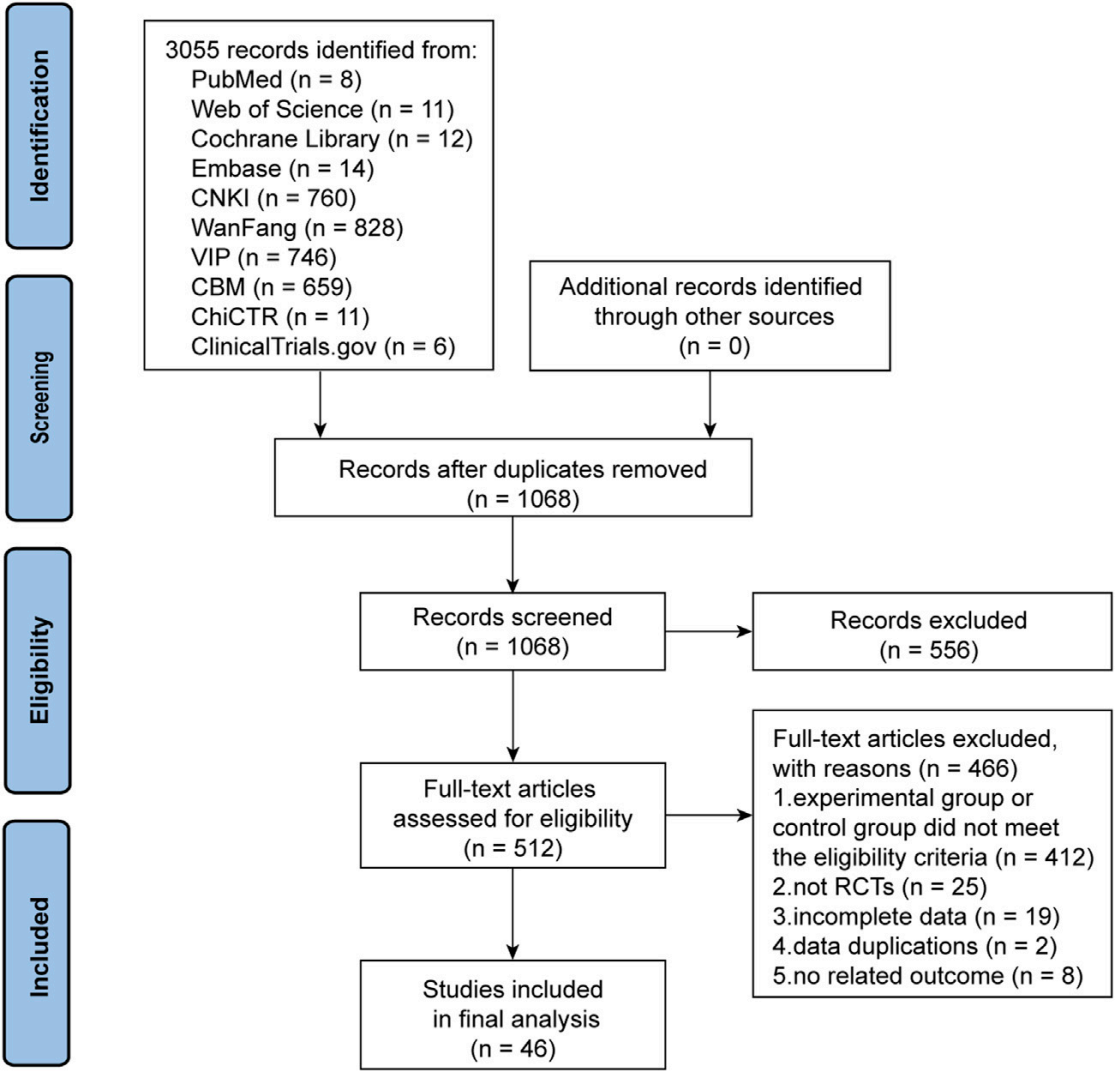
The PRISMA flow chart of the literature screening and selection process.

We initially identified 3,055 articles with potential relevance, of which 1,987 duplicates were excluded. Of the remaining 1,068 articles, 556 were excluded after reviewing their titles and abstracts. The remaining articles were further evaluated by reading the full text, and 465 were removed for the following reasons: the experimental or control group met the exclusion criteria (n = 466), not an RCT (n = 25), incomplete data (n = 19), duplicate data (n = 2), and lacking outcomes that met the criteria (n = 8). Finally, 46 trials were included in the quality assessment and further analysis. Figure 1 presents the PRISMA flowchart illustrating the trial selection process.

### 3.2 Study characteristics

The 46 included RCTs were performed from 2007 to 2022. One RCT was a multicenter study. The sample size varied from 60 to 182, totaling 4,601 participants (2,330 and 2,271 in the experimental and control groups, respectively). The participants were predominantly male and ranged from 45.81 to 75.8 years old. All the research interventions included DHI combined with CWM. In all studies, DHI was intravenously administered at a dose of 20–40 mL/day for 7–30 days. All the included studies provided comprehensive reports on the outcomes of inflammatory factors and vascular endothelial function. Furthermore, 21 trials reported AE outcomes, of which 11 reported no AEs, and the remaining 10 reported 70 AEs. Table 1 summarizes the information regarding the included RCTs.

**TABLE 1:** Characteristics of the included RCTs and the detail of PICOS.

Study ID	Samplesize (T/C)	Age (T/C)	Male (%) (T/C)	Interventions		Duration	Outcome
				Trial group	Control group		
Gong and Li (2007)	45/35	65.00 ± 6.21/66.00 ± 7.28	24(53.33%)/18(51.43%)	DHI 20 mg qd + CWM	CWM	14 days	④⑤
Feng et al. (2008)	30/30	unclear	unclear	DHI 20 mg qd + CWM	CWM	14 days	②③
Liao et al. (2008)	40/30	65.10 ± 13.50/64.20 ± 10.30	19(47.50%)/17(56.67%)	DHI 20 mg qd + CWM	CWM	10 days	①⑥
Chang et al. (2009)	80/80	unclear	unclear	DHI 30 mg qd + CWM	CWM	14 days	④
Gao et al. (2009)	42/40	unclear	unclear	DHI 20 mg qd + CWM	CWM	14 days	①⑤⑦
Hu and Chen (2009)	32/31	unclear	unclear	DHI 30 mg qd + CWM	CWM	14 days	①
Li et al. (2009)	92/90	61.00 ± 14.00/63.00 ± 11.00	55(59.78%)/54(60.00%)	DHI 20 mg qd + CWM	CWM	14 days	①
Shi (2009)	60/60	62.90 ± 6.10/63.10 ± 7.00	46(76.67%)/47(78.33%)	DHI 30 mg qd + CWM	CWM	14 days	①③
Yang and Shi (2009)	60/60	62.90 ± 6.90/63.10 ± 7.20	46(76.67%)/47(78.33%)	DHI 30 mg qd + CWM	CWM	14 days	①③
Zhang et al. (2009)	62/58	63.00 ± 12.00/61.00 ± 11.00	38(61.29%)/35(60.34%)	DHI 20 mg qd + CWM	CWM	14 days	②③⑦
Zhang (2010)	58/58	unclear	unclear	DHI 40 mg qd + CWM	CWM	14 days	①
Hu and Liu (2011)	36/36	unclear	unclear	DHI 30 mg qd + CWM	CWM	14 days	①②④⑤
Jia (2011)	35/35	unclear	unclear	DHI 30 mg qd + CWM	CWM	14 days	①⑦
Luo and Huang (2011)	39/39	unclear	unclear	DHI 20 mg qd + CWM	CWM	14 days	④⑤
Sun et al. (2011)	51/51	65.61 ± 5.82/66.10 ± 7.27	35(68.63%)/31(60.78%)	DHI 20 mg qd + CWM	CWM	14 days	①⑦
Xie and Cao (2011)	32/32	62.80 ± 8.20/63.00 ± 8.50	20(62.50%)/18(56.25%)	DHI 30 mg qd + CWM	CWM	14 days	①⑦
Zhao et al. (2011)	36/36	50.90 ± 8.60/60.20 ± 9.20	unclear	DHI 40 mg qd + CWM	CWM	14 days	①⑦
Gong (2012)	45/45	56.04 ± 14.35/55.70 ± 12.01	22(48.89%)/24(53.33%)	DHI 40 mg qd + CWM	CWM	84 days	④⑤⑦
Han (2012)	32/32	unclear	unclear	DHI 20 mg qd + CWM	CWM	14 days	①⑦
Liu et al. (2012)	48/48	62.50 ± 3.60/62.90 ± 3.10	27(56.25%)/25(52.08%)	DHI 40 mg qd + CWM	CWM	15 days	①⑦
Shi and Liu (2012)	80/76	unclear	40(50.00%)/39(51.32%)	DHI 40 mg qd + CWM	CWM	14 days	①
Xu and Min (2012)	46/46	70.40/68.70	26(56.52%)/25(54.35%)	DHI 30 mg qd + CWM	CWM	15 days	①⑦
Liu and Wang (2013)	40/40	47.30/48.80	23(57.50%)/25(62.50%)	DHI 30 mg qd + CWM	CWM	10 days	①
Pan et al. (2013)	63/63	unclear	unclear	DHI 30 mg qd + CWM	CWM	28 days	④⑤⑦
Chen J. et al. (2015)	94/73	64.83 ± 8.45/64.54 ± 9.64	59(62.77%)/47(64.38%)	DHI 30 mg qd + CWM	CWM	14 days	①②③
Chen S. et al. (2015)	65/65	68.00 ± 12.00/69.00 ± 14.00	43(66.15%)/49(75.38%)	DHI 30 mg qd + CWM	CWM	14 days	①
Fu and Jiang (2015)	49/47	46.17 ± 5.43/45.81 ± 4.79	27(55.10%)/26(55.32%)	DHI 30 mg qd + CWM	CWM	10 days	①
Kang and Hou (2015)	60/60	62.50 ± 5.40/62.80 ± 6.10	32(53.33%)/29(48.33%)	DHI 40 mg qd + CWM	CWM	14 days	①
Liu et al. (2015)	52/52	61.50 ± 9.10/63.80 ± 9.90	32(61.54%)/34(65.38%)	DHI 30 mg qd + CWM	CWM	14 days	①
Ma (2015)	40/40	56.41 ± 4.52/55.64 ± 4.23	24(60.00%)/23(57.50%)	DHI 20 mg qd + CWM	CWM	14 days	①⑥
Zhao et al. (2015)	54/54	61.93 ± 4.41/62.53 ± 4.73	27(50.00%)/26(48.15%)	DHI 30 mg qd + CWM	CWM	14 days	①②④⑤
Li et al. (2016)	43/43	unclear	unclear	DHI 20–40 mg bid + CWM	CWM	14 days	①②③⑦
Lv (2016)	65/65	62.40 ± 3.70/64.40 ± 4.00	38(58.46%)/34(52.31%)	DHI 20 mg qd + CWM	CWM	15 days	①⑦
Yang et al. (2016)	34/31	63.47 ± 9.83/62.93 ± 9.49	21(61.76%)/19(61.29%)	DHI 40 mg qd + CWM	CWM	10 days	⑥⑦
Wang (2017)	45/45	73.26 ± 2.47/73.38 ± 2.52	26(57.78%)/21(46.67%)	DHI 20 mg qd + CWM	CWM	14 days	④⑤
Zhang et al. (2017)	50/50	60.00 ± 7.00/58.00 ± 7.00	30(60.00%)/28(56.00%)	DHI 30 mg qd + CWM	CWM	7 days	①②③
Li (2018)	40/39	60.25 ± 8.31/60.33 ± 9.14	31(77.50%)/29(74.36%)	DHI 20–40 mg bid + CWM	CWM	28 days	④⑤⑦
Li and Zhang (2018)	60/60	75.80 ± 4.50/75.20 ± 4.90	36(60.00%)/35(58.33%)	DHI 40 mg qd + CWM	CWM	30 days	⑤⑦
Lu et al. (2018)	36/36	65.79 ± 5.83/64.25 ± 6.57	20(55.56%)/22(61.11%)	DHI 30 mg qd + CWM	CWM	14 days	①②③⑦
Wang (2018)	41/41	58.00 ± 6.00/58.00 ± 6.00	17(41.46%)/15(36.59%)	DHI 40 mg qd + CWM	CWM	14 days	⑥
Yin et al. (2018)	40/40	69.30 ± 9.20/67.30 ± 6.40	18(45.00%)/19(47.50%)	DHI 30 mg qd + CWM	CWM	14 days	①⑥⑦
Yu (2018)	50/50	61.63 ± 3.16/61.47 ± 3.20	28(56.00%)/27(54.00%)	DHI 30 mg qd + CWM	CWM	28 days	②③⑦
Duan (2019)	63/63	63.25 ± 3.47/62.63 ± 3.58	34(53.97%)/35(55.56%)	DHI 30 mg qd + CWM	CWM	14 days	⑥
Yu and Xu (2021)	36/36	57.20 ± 13.20/56.80 ± 12.10	25(69.44%)/24(66.67%)	DHI 30 mg qd + CWM	CWM	14 days	②③⑦
Zhang (2021)	50/50	59.75 ± 4.05/59.73 ± 4.02	29(58.00%)/28(56.00%)	DHI 20 mg qd + CWM	CWM	14 days	①⑥
Chen et al. (2022)	79/80	61.22 ± 8.08/60.25 ± 7.51	54(68.35%)/44(55.00%)	DHI 40 mg qd + CWM	CWM + placebo	7 days	①⑦

T/C, Trial group/Control group; DHI, Danhong injection; CWM, conventional western medicine; ①hs-CRP: high-sensitivity C-reactive protein; ②TNF-α: tumor necrosis factor-α; ③IL-6: interleukin-6; ④NO: nitric oxide; ⑤ET/ET-1: endothelin/endothelin-1; ⑥Hcy: homocysteine; ⑦AEs: adverse events.

### 3.3 Risk of bias

**FIGURE 2:**
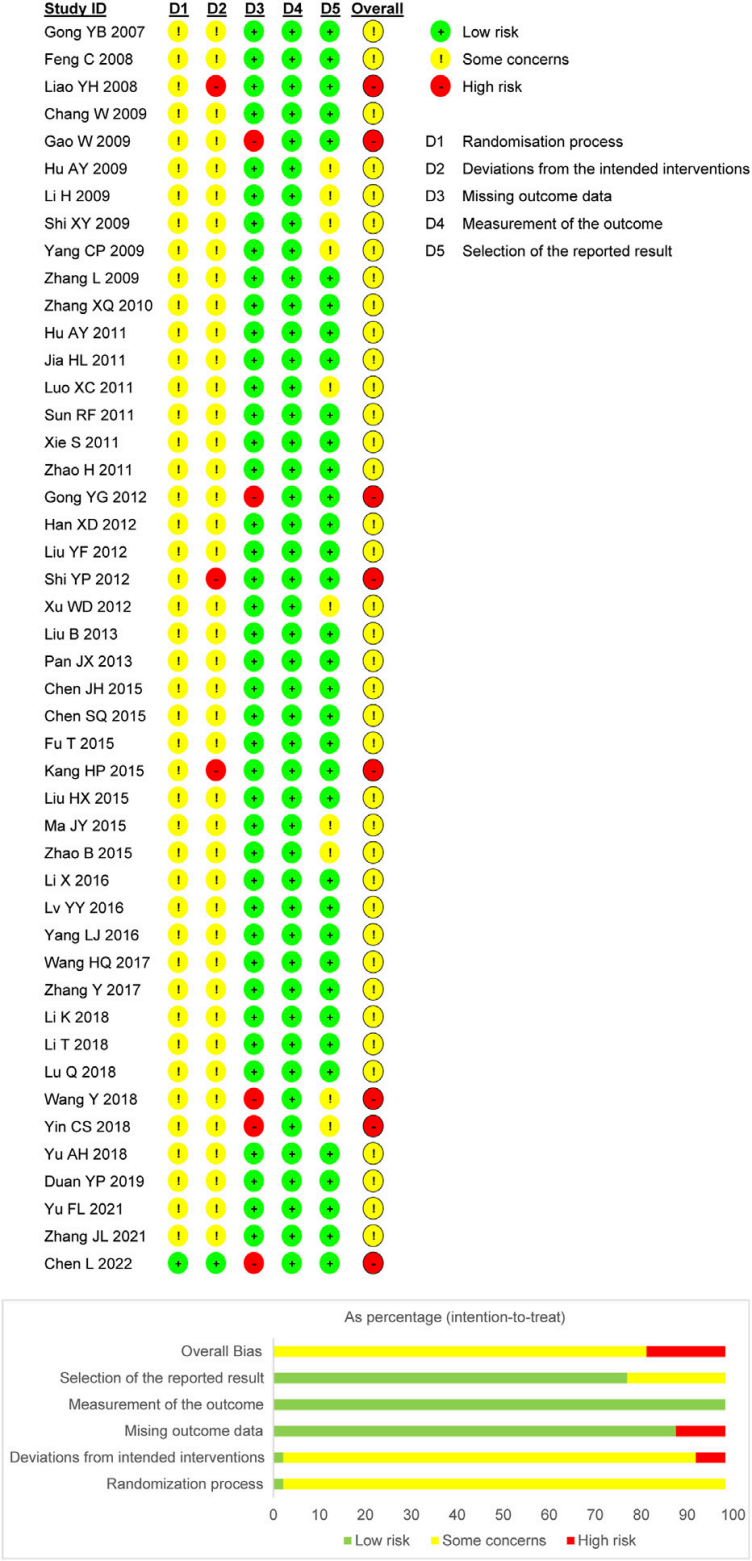
Risk of bias assessment for included RCTs.

Nine trials utilized randomized sequence methods, such as random number tables or central randomized systems. Of these, 1 trial mentioned allocation concealment methods and was therefore considered “low risk.” The remaining trials only mentioned randomization and did not propose specific randomization methods; therefore, the risk of bias was unclear. For bias due to deviations from the expected interventions, 3 trials were identified as “high risk” due to the absence of blinding, and 1 trial was classified as “low risk” for explicitly implementing blinding. The risk in the remaining trials was unclear due to the lack of blinding information regarding the participants, operators, and outcome assessments. The absence of follow-up was not explicitly addressed in five trials; thus, bias due to missing outcome data was assessed as “high risk.” Moreover, all trials were rated as “high risk” for bias owing to the objectivity of the outcome measures. In addition, 10 trials raised “some concern” regarding potential bias in the selection of reported outcomes due to the absence of explicitly mentioned planned outcomes in the predetermined protocol. However, the remaining trials were considered “low risk,” as they consistently adhered to the predetermined protocol regarding outcome measures and analyses. Based on the assessment of these five aforementioned aspects, 8 trials were classified as “high risk,” while the remaining trials were classified as “some concern.” Figure 2 presents the risk of bias evaluation results.

### 3.4 Inflammatory factors

#### 3.4.1 hs-CRP

**FIGURE 3:**
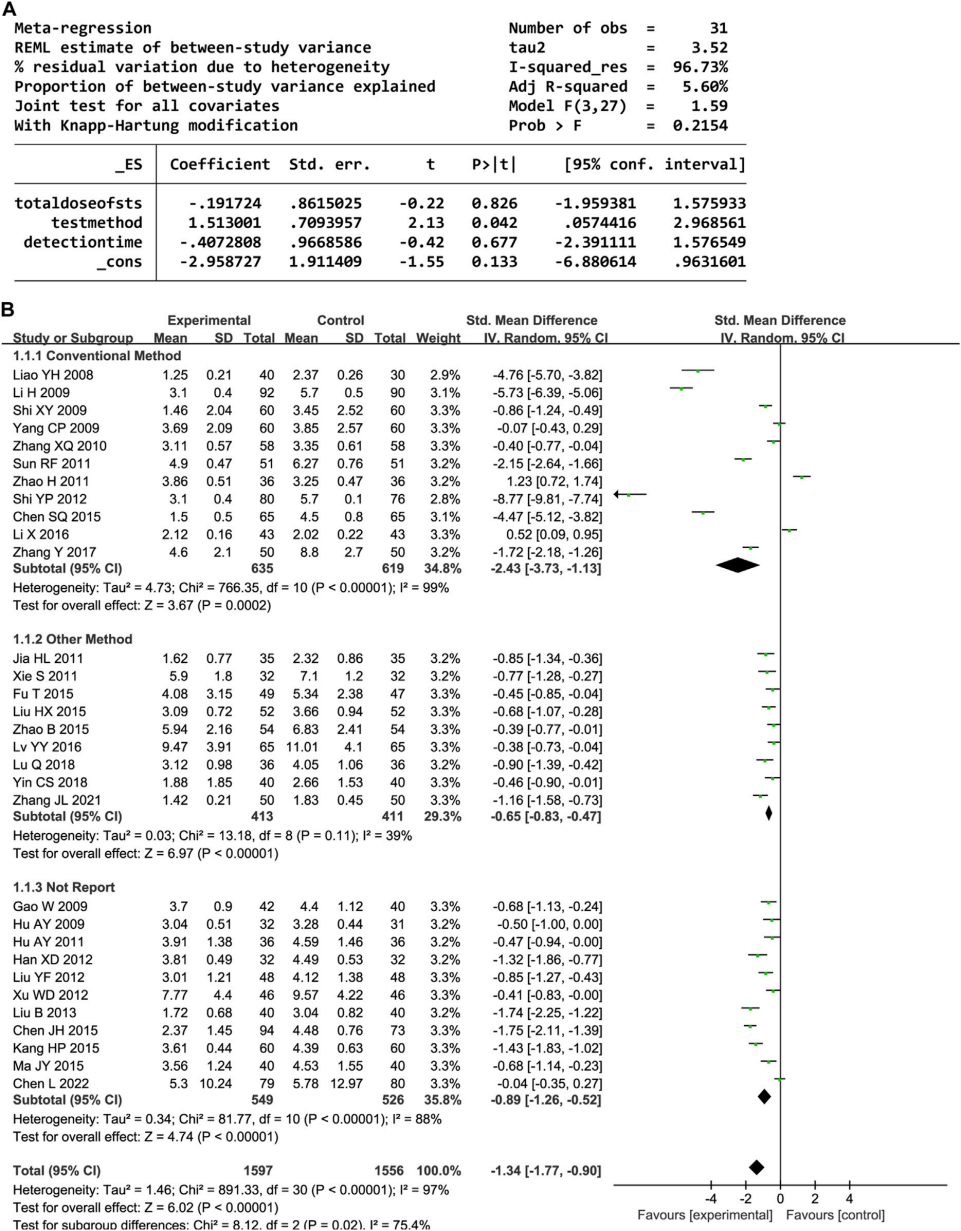
**(A)** Meta-regression results for hs-CRP. **(B)** Forest plot of hs-CRP.

In total, 31 trials documented the serum hs-CRP level. A random-effects model was employed owing to significant heterogeneity (P < 0.00001, I^2^ = 97%). The results indicated notably decreased serum hs-CRP levels in the experimental group compared to the control group (SMD = −1.34, 95% confidence interval [CI] [-1.77, −0.90], P < 0.00001). A sensitivity analysis was conducted owing to the heterogeneity, but none of the studies were substantially related to the estimated effect sizes (Supplementary Figure S1). A meta-regression was conducted to explore potential factors contributing to the observed heterogeneity, which identified an association with the hs-CRP test method (P < 0.05, Figure 3A). A subgroup analysis based on the test method resulted in decreased heterogeneity (Figure 3B).

#### 3.4.2 TNF-α

**FIGURE 4:**
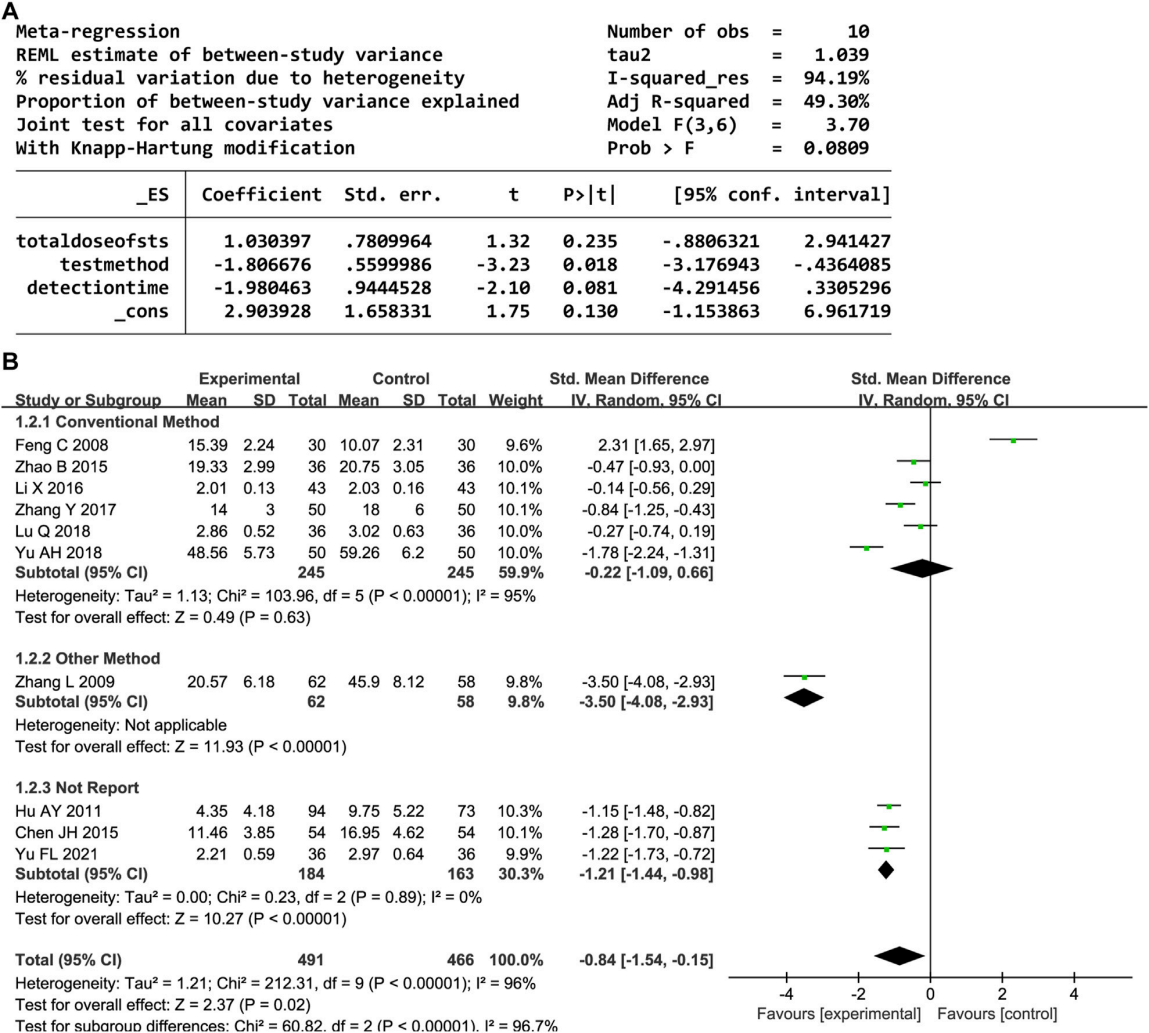
**(A)** Meta-regression results for TNF-α. **(B)** Forest plot of TNF-α.

Overall, 10 trials reported the serum TNF-α level. A random-effects model was employed owing to significant heterogeneity (P < 0.00001, I^2^ = 96%). DHI with CWM significantly reduced the TNF-α level compared to the control group (SMD = −0.84, 95% CI [-1.54, −0.15], P = 0.02). A sensitivity analysis was performed owing to the heterogeneity, but none of the studies were significantly associated with the estimated effect sizes (Supplementary Figure S2). The meta-regression analysis identified an association between the test method and heterogeneity (P < 0.05, Figure 4A); the detection time and total DHI dose had no effect. A subgroup analysis based on the test method resulted in decreased heterogeneity (Figure 4B).

#### 3.4.3 IL-6

**FIGURE 5:**
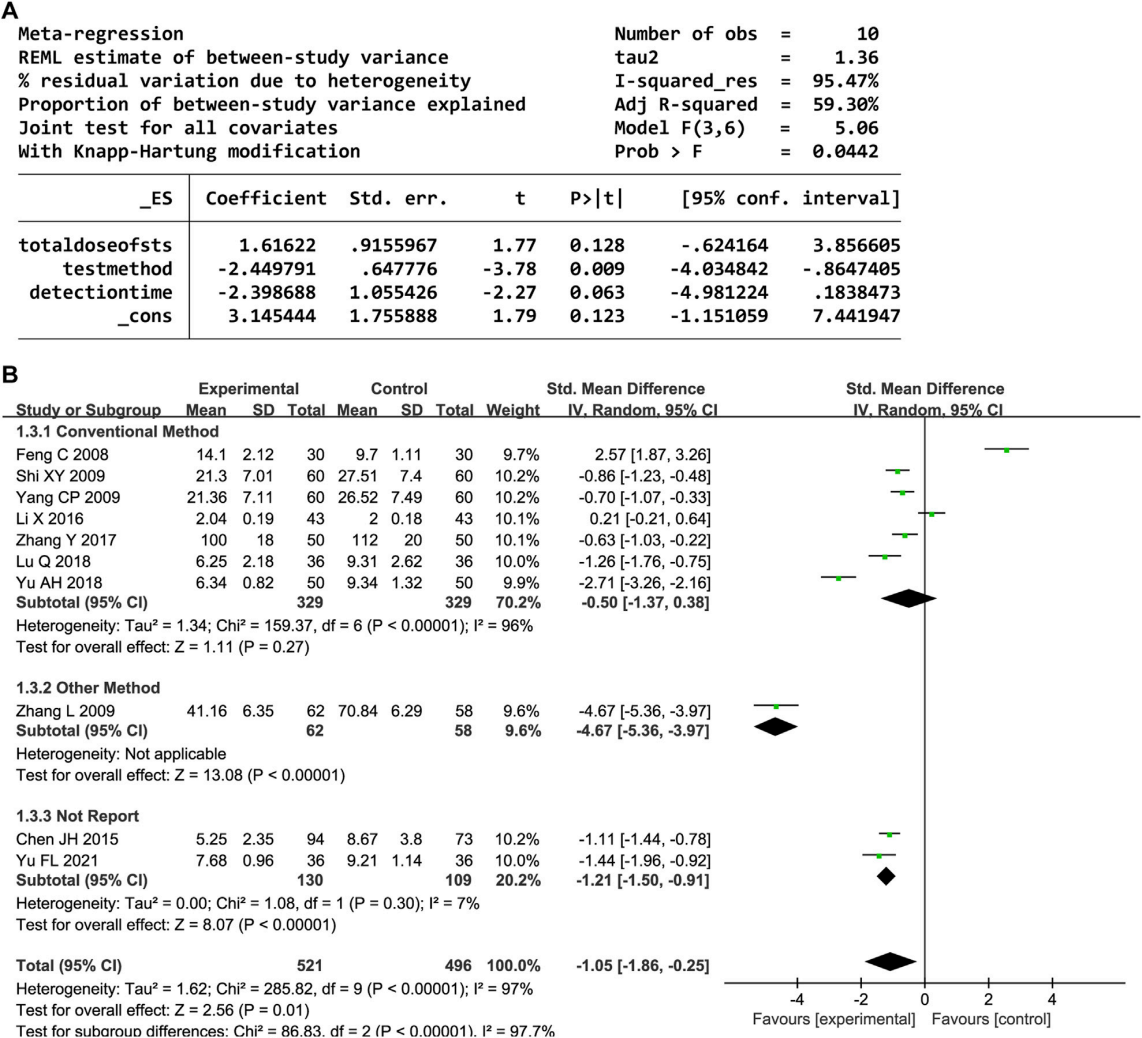
**(A)** Meta-regression results for IL-6. **(B)** Forest plot of IL-6.

Ten trials provided data on the serum IL-6 level. Significant heterogeneity was observed (P < 0.00001, I^2^ = 97%); thus, a random-effects model was employed. The IL-6 levels significantly decreased in the DHI group compared to the control group (SMD = −1.05, 95% CI [-1.86, −0.25], P = 0.01). The sensitivity analysis showed that no one study affected the summary effect (Supplementary Figure S3). The meta-regression analysis identified an association between the heterogeneity and IL-6 test method (P < 0.05, Figure 5A). A subgroup analysis based on the test method resulted in decreased heterogeneity (Figure 5B).

### 3.5 Vascular endothelial function

#### 3.5.1 NO

**FIGURE 6:**
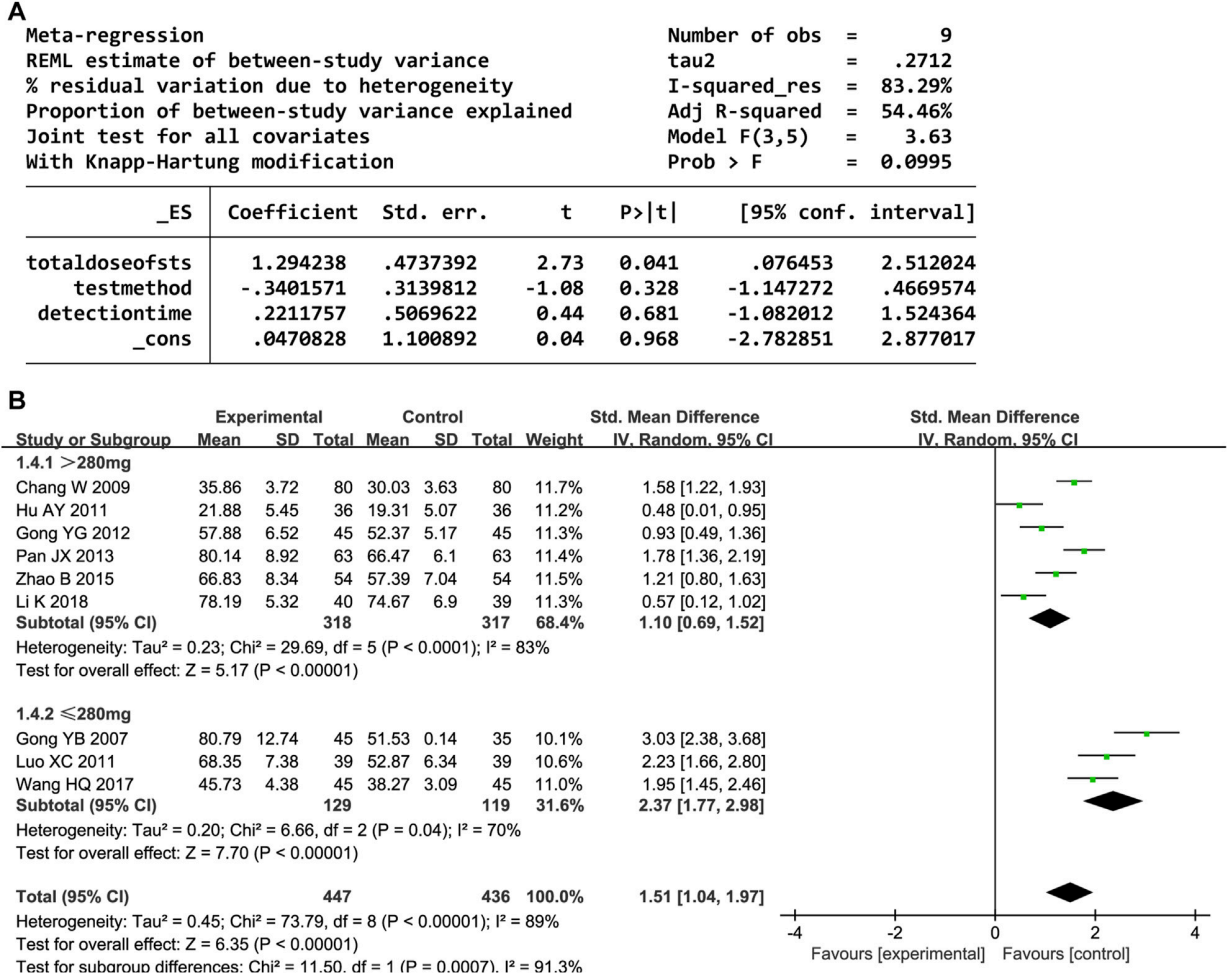
**(A)** Meta-regression results for NO. **(B)** Forest plot of NO.

Nine trials reported the NO level. The random-effects model was used owing to high heterogeneity (P < 0.00001, I^2^ = 89%). The NO level significantly increased after administering DHI (SMD = 1.51, 95% CI [1.04, 1.97], P < 0.00001). The sensitivity analyses showed that no one study affected the estimated effect sizes (Supplementary Figure S4). The meta-regression analysis showed that heterogeneity and detection methods were related to the total DHI dose (P < 0.05; Figure 6A). Further subgroup analyses revealed decreased heterogeneity (Figure 6B).

#### 3.5.2 ET/ET-1

**FIGURE 7:**
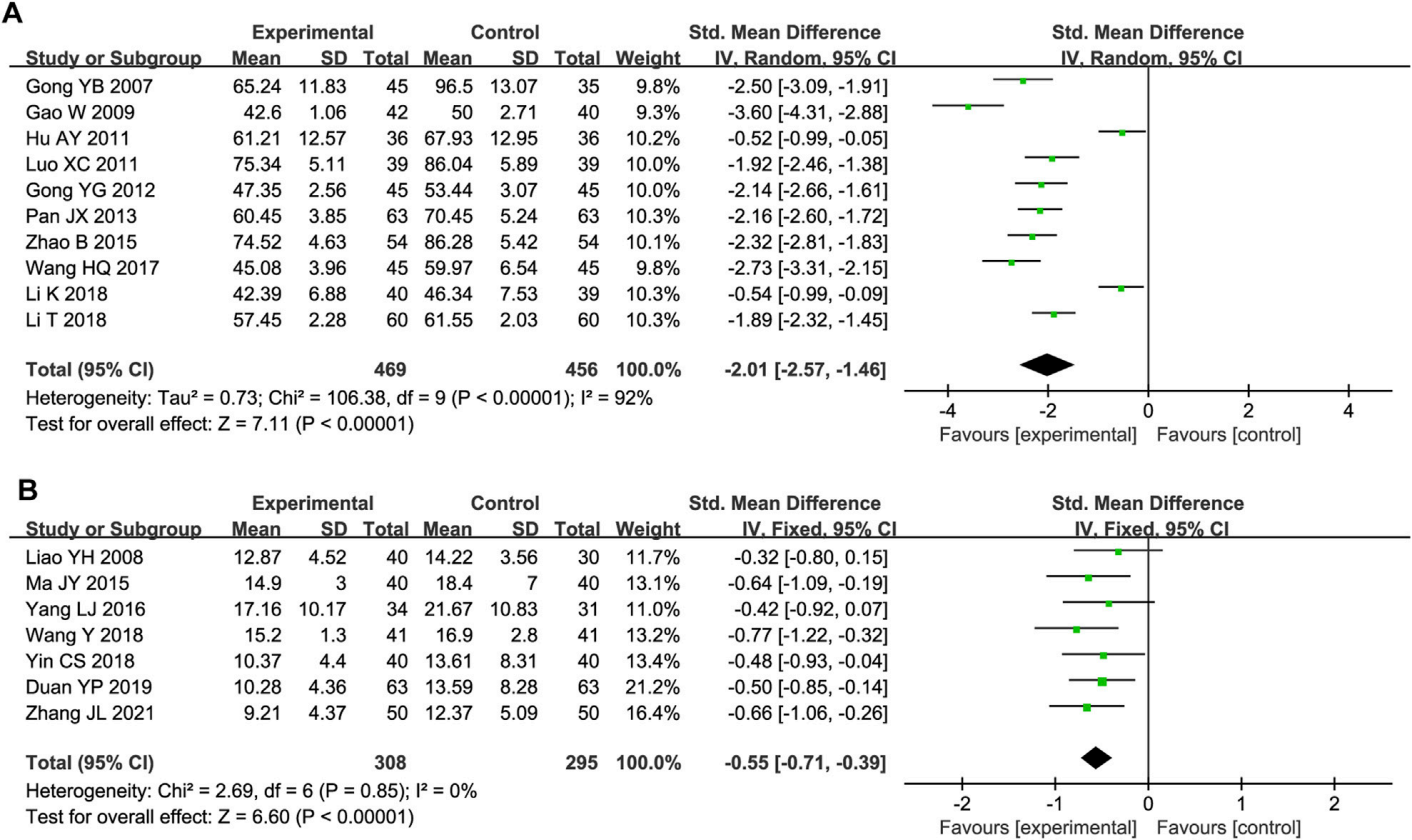
**(A)** Forest plot of ET/ET-1. **(B)** Forest plot of Hcy.

Ten studies included serum ET/ET-1 level data. Substantial heterogeneity was observed (P < 0.00001, I^2^ = 92%); thus, a random-effects model was used. The ET/ET-1 level was significantly lower in the experimental group than in the control group (SMD = −2.01, 95% CI [-2.57, −1.46], P < 0.00001; Figure 7A). The sensitivity analysis demonstrated the robustness and reliability of the results (Supplementary Figure S5). A meta-regression was conducted to assess how the detection time, total DHI dose, and test method influenced the heterogeneity but did not reveal the source (Supplementary Figure S6).

#### 3.5.3 Hcy

Seven trials reported Hcy outcomes. The fixed-effect model was employed owing to the absence of heterogeneity (P = 0.85, I^2^ = 0%). DHI treatment decreased the Hcy level more effectively than the control treatment (SMD = −0.55, 95% CI [-0.71, −0.39], P < 0.00001; Figure 7B).

### 3.6 AEs

**FIGURE 8:**
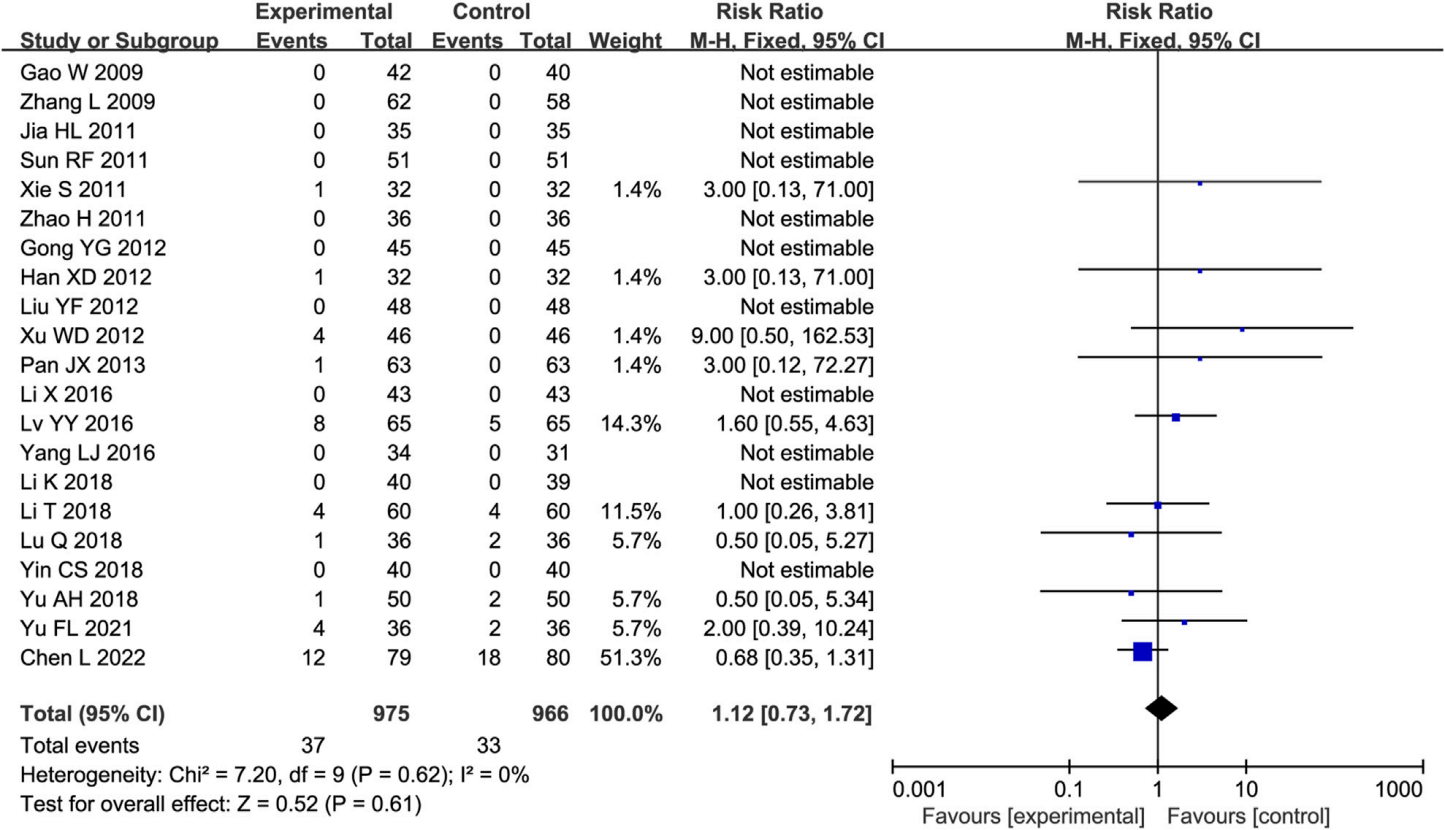
Forest plot of adverse events (AEs).

Twenty-one trials were analyzed for AEs, of which 10 reported no AEs in either group. The main AEs were gastrointestinal reactions, dizziness or headache, and palpitations (Supplementary Table S2). No substantial heterogeneity was identified (P = 0.61, I^2^ = 0%); thus, a fixed-effects model was employed. The AE incidence rates were comparable between the experimental and control groups (RR = 1.12, 95% CI [0.73, 1.72], P = 0.61; Figure 8).

### 3.7 Publication bias

**FIGURE 9:**
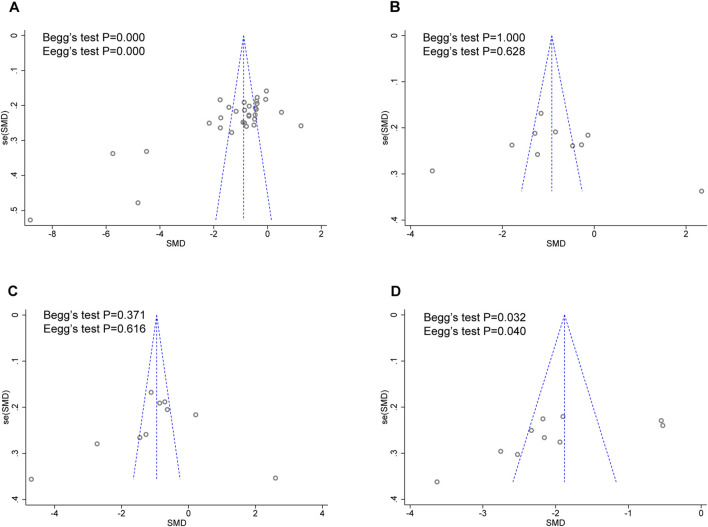
Funnel plot for publication bias assessment. **(A)** hs-CRP publishes biased assessment. **(B)** TNF-α publishes biased assessment. **(C)** IL-6 publishes biased assessment. **(D)** ET/ET-1 publishes biased assessment.

As there were over ten studies reported hs-CRP, TNF-α, IL-6, and ET/ET-1 levels, we performed Egger’s and Begg’s tests to evaluate the potential impact of publication bias in these studies (Figure 9). The results showed that except for hs-CRP and ET/ET-1, there was no possibility of publication bias in the other two groups. Although the statistical significance of publication bias was negligible, we considered it more likely because all but one of the included trials were conducted in China.

### 3.8 GRADE assessment

The GRADE evaluation system was utilized to evaluate the level of certainty in the evidence regarding the outcomes. The quality of evidence ranged from “very low” to “low.” The primary factors contributing to this downgrade were a high risk of bias, imprecision, and inconsistency. The results from the comprehensive GRADE evaluation can be found in Supplementary Table S3.

## 4 Discussion

The present systematic review analyzed 46 RCTs involving 4,601 participants to evaluate the impact of DHI on inflammatory factors and vascular endothelial function in individuals diagnosed with UAP. Treatment with DHI significantly reduced the hs-CRP, TNF-α, IL-6, ET/ET-1, and Hcy levels and increased the NO level. Furthermore, the AE incidence rates did not differ between the DHI and control groups. Some of the patients with UAP in the included RCTs had comorbid chronic metabolic diseases, such as hypertension, diabetes, and hyperlipidemia. Although this aspect was not evaluated in this study, many others have shown that DHI improves metabolic markers (Chen et al., 2014; Fan et al., 2018; Orgah et al., 2020; Yang et al., 2024), which is also conducive to improving UAP. Therefore, DHI as a supplementary treatment enhances the patient’s prognosis by safely and efficiently diminishing inflammatory factors and promoting endothelial function among those with UAP.

Previous studies have predominantly focused on the effects of DHI on myocardial injury and angina in patients with UAP. To our knowledge, this systematic review is the first to extensively focus on how DHI treatment affects inflammatory factors and vascular endothelial function among individuals diagnosed with UAP. UAP is a progressive disease involving multiple immune cells and inflammatory factors, and imbalanced inflammatory responses and endothelial dysfunction might play a crucial role in the formation, growth, and plaque rupture of atherosclerosis in these patients (Wirtz and von Känel, 2017; Fioranelli et al., 2018). The two promote each other through complex molecular mechanisms, such as pro-inflammatory factors (e.g., TNF-α and IL-6) that reduce NO production by inhibiting endothelial NO synthase while activating ET-1 release and oxidative stress products (e.g., malondialdehyde) that accelerate endothelial dysfunction through the degradation of NO, creating a vicious cycle of inflammation and endothelial damage (Gupta et al., 2020).

We also found that DHI treatment significantly reduced the hs-CRP, TNF-α, and IL-6 levels (P < 0.05). hs-CRP is a vascular inflammation marker and a predictor of myocardial infarction (Li Y. et al., 2022). Moreover, TNF-α and IL-6 are important pro-inflammatory factors that promote the accumulation of immune complexes in endothelial cells, which increase the risk of thrombosis and participate in the vascular inflammatory response and coronary atherosclerosis (Gao et al., 2018; Bi et al., 2019). DHI treatment reduces TNF-α levels in patients with UAP, which can lead to the decreased expression of monocyte chemoattractant protein-1 and suppress the inflammatory response (Ren et al., 2010).

Moreover, we found that DHI treatment significantly reduced the ET/ET-1 and Hcy levels and increased the NO level (P < 0.05). NO is a key vasodilator secreted by endothelial cells and a central regulator of vascular endothelial function (Bilsborough et al., 2003). ET-1 is a subtype of ET that is released by endothelial cells during acute and chronic vascular injury and is the most potent vasoconstrictor (Caroselli et al., 2016). NO and ET-1 maintain a dynamic equilibrium in healthy vessels and work together to regulate vascular tone. However, in atherosclerotic plaques, the pro-inflammatory effects of ET-1, together with decreased NO, accelerate disease progression (Kageyama et al., 2014). Hcy impairs vascular endothelial function through various mechanisms, such as interfering with NO metabolism and activating endoplasmic reticulum stress and epigenetic regulation, leading to endothelial activation, oxidative stress, and inflammatory responses and accelerating vascular pathology. Elevated Hcy levels in the bloodstream are also a risk factor for the development of atherosclerosis (Guo et al., 2009).

The most common AEs in the included RCTs were gastrointestinal reactions, including nausea, vomiting, abdominal pain, and flatulence, which may result from the drug’s normal pharmacologic effects (Li et al., 2015). Although some trials reported AEs, they did not yield fully accurate results considering that DHI administration was combined with CWM; thus, evidence regarding whether DHI was directly linked to the symptoms was lacking. In addition, some trials did not report AEs. Considering that adverse reactions do not fully assess drug safety outcomes, more rigorous safety and toxicity assessments are necessary to substantiate the evidence for further consideration.

Studies suggest that DHI treatment is more clinically effective against UAP compared to traditional Western medicine. Compared with two previous meta-analyses (Xu et al., 2011; Liu et al., 2016), this systematic review provides a more comprehensive assessment analyzing DHI’s ability to reduce inflammatory factors and improve vascular endothelial function in patients with UAP. The prior studies only included hs-CRP as an inflammation indicator, whereas this study incorporated newly published and well-conducted RCTs with more comprehensive outcome measures. This study also synthesized inter-study heterogeneity using sensitivity, meta-regression, and subgroup analyses, which have not been explored in previous systematic evaluations. A prior network meta-analysis (Li M. et al., 2022) explored the comparative efficacy of different DHIs for UAP, reporting that DHI was superior to other Danshen preparations in terms of overall efficacy and hs-CRP improvements, aligning with our findings. Together, these results suggest that DHI improves inflammation in patients with UAP, but these improvements in clinical symptoms and lipid levels may vary based on the Danshen preparation. Finally, several clinical trials have reported that DHI promotes vascular endothelial repair (Hu et al., 2016), which could be another future research direction.

## 5 Limitations

Before applying the results of this study in clinical practice, it is crucial to recognize specific constraints that could impact its practicality. First, the quality of the 46 included trials was “very low” to “low,” and there was a lack of high-quality trials rigorously designed under the CONSORT protocol. Second, a significant number of trials lacked explicit methodological information, such as randomization protocols, concealment of allocation, and blinding techniques. As a result, the reliability of the evidence presented was substantially compromised. In conclusion, it is crucial to approach the results cautiously due to the presence of substantial heterogeneity and potential publication bias.

## 6 Conclusion

DHI treatment effectively and safely reduced the hs-CRP, TNF-α, IL-6, ET/ET-1, and Hcy levels and increased the NO level in patients with UAP. However, considering the overall low quality of the original studies, further large-scale, high-quality RCTs are imperative to provide robust evidence for clinical practice.

## Data Availability

The original contributions presented in the study are included in the article/Supplementary Material, further inquiries can be directed to the corresponding author.
