# Supplementary material for: Efficacy and safety of Danhong injection on inflammatory factors and vascular endothelial function in patients with unstable angina pectoris: a systematic review and meta-analysis of randomized clinical trials
Source: Front Pharmacol. 2025 Jun 13;16:1389746. doi: 10.3389/fphar.2025.1389746 (PMC12202559; doi:10.3389/fphar.2025.1389746)
Supplement: Supplementary file 1 [file DataSheet1.docx]

Supplementary Material

## Supplementary Figures


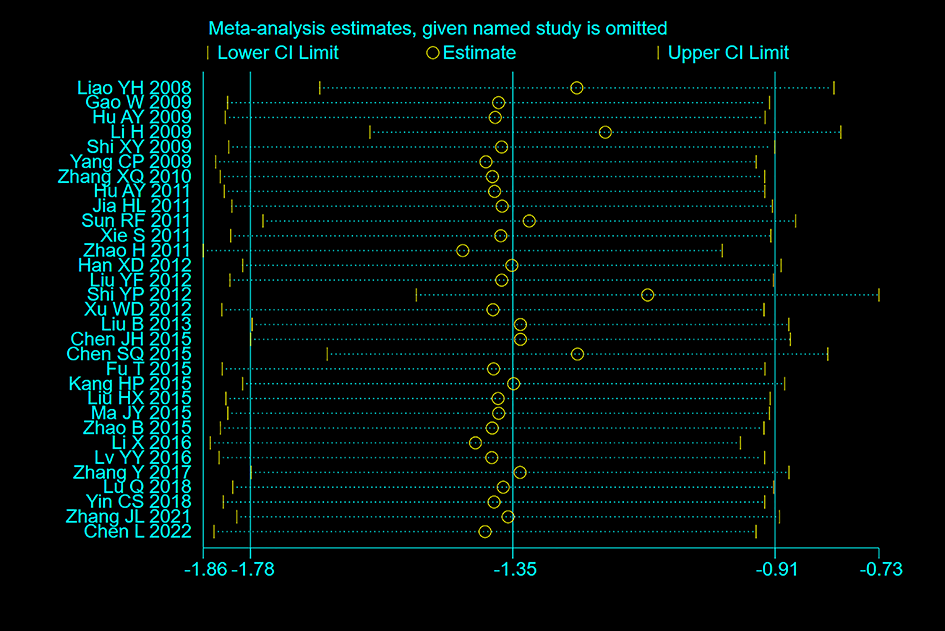


**Fig. S1.** Sensitivity analysis of hs-CRP.


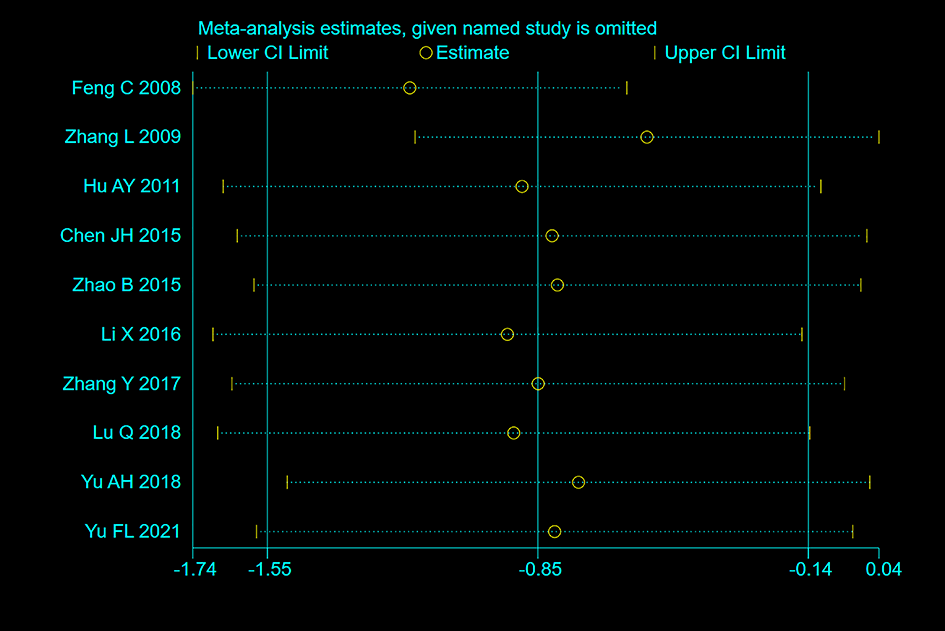


**Fig. S2.** Sensitivity analysis of TNF-α.


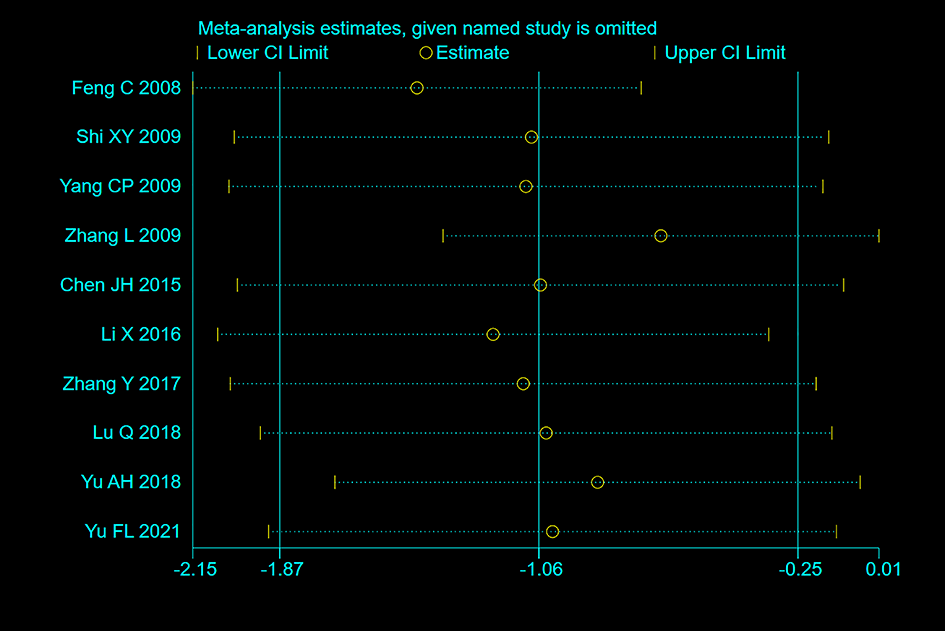


**Fig. S3.** Sensitivity analysis of IL-6.


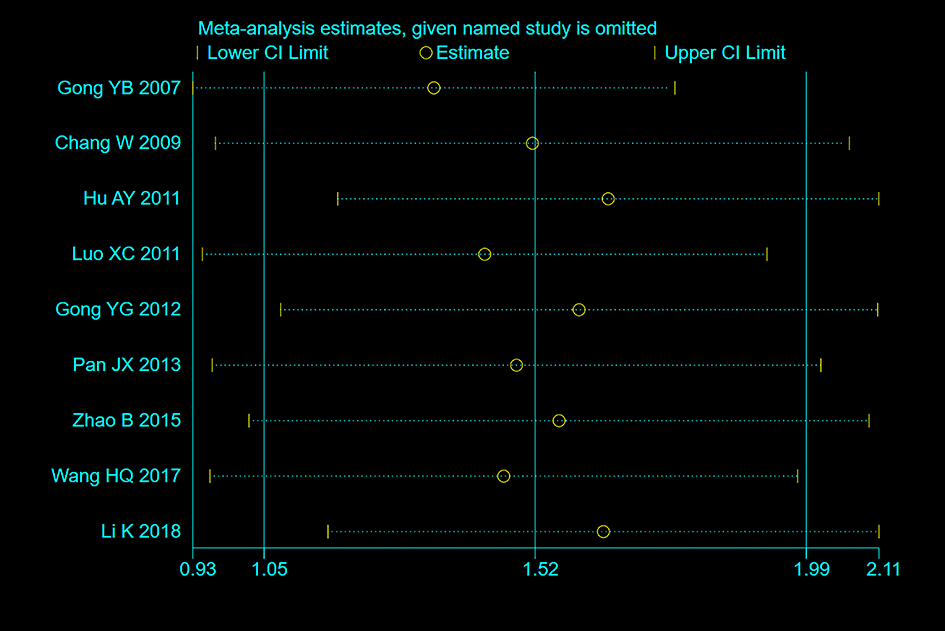


**Fig. S4.** Sensitivity analysis of NO.


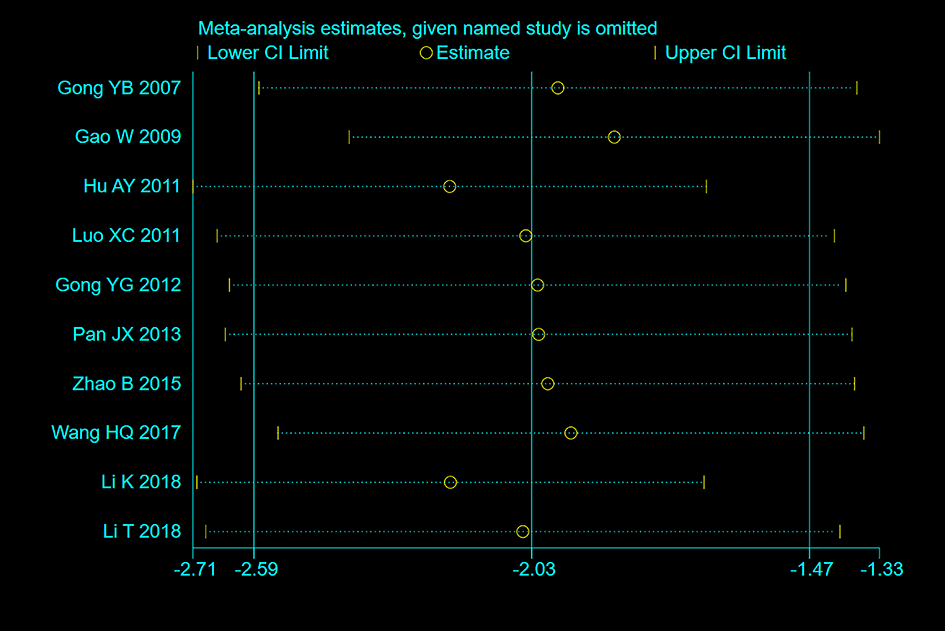


**Fig. S5.** Sensitivity analysis of ET/ET-1.

**
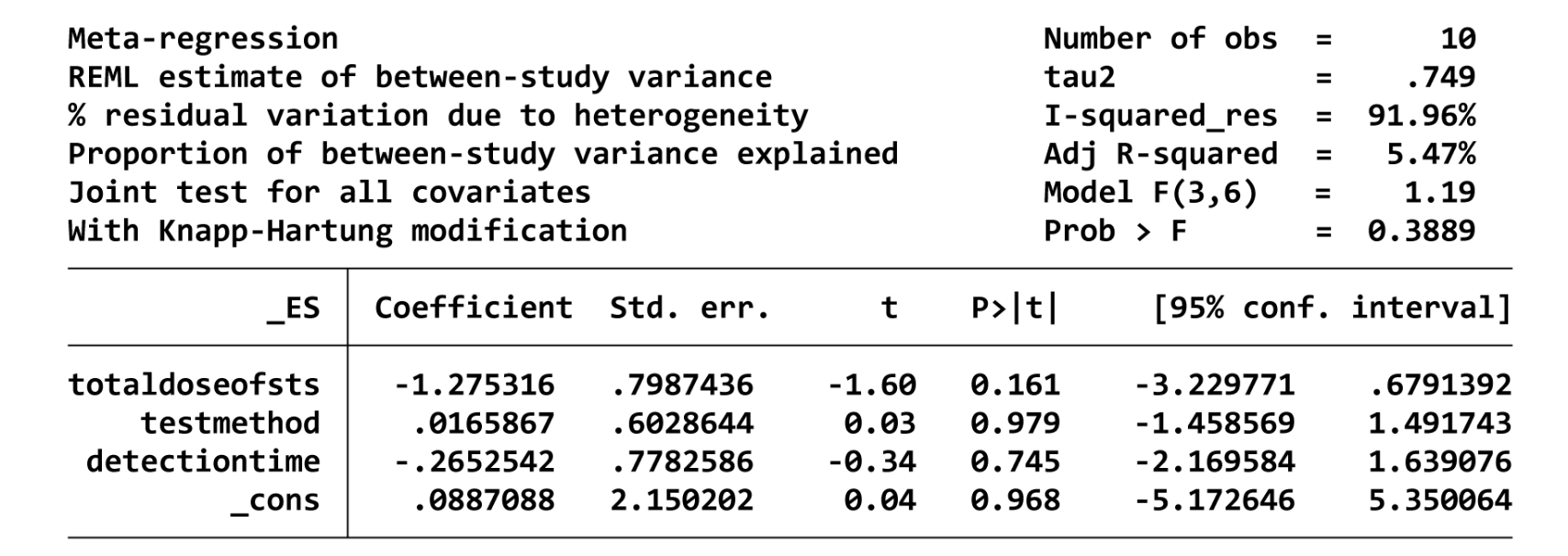
**

**Fig. S6.** Meta-regression analysis of ET/ET-1.

- 1. **Supplementary Tables**

| **Databases** | **Search items** | |
| --- | --- | --- |
| Pubmed | #1 | "Angina, Unstable"[Mesh] |
|  | #2 | unstable angina[Title/Abstract] |
|  | #3 | (((((((((((((((((Anginas, Unstable[Title/Abstract]) OR (Unstable Anginas[Title/Abstract])) OR (Angina Pectoris, Unstable[Title/Abstract])) OR (Angina Pectori, Unstable[Title/Abstract])) OR (Unstable Angina Pectori[Title/Abstract])) OR (Unstable Angina Pectoris[Title/Abstract])) OR (Unstable Angina[Title/Abstract])) OR (Angina at Rest[Title/Abstract])) OR (Angina, Preinfarction[Title/Abstract])) OR (Anginas, Preinfarction[Title/Abstract])) OR (Preinfarction Angina[Title/Abstract])) OR (Preinfarction Anginas[Title/Abstract])) OR (Myocardial Preinfarction Syndrome[Title/Abstract])) OR (Myocardial Preinfarction Syndromes[Title/Abstract])) OR (Preinfarction Syndrome, Myocardial[Title/Abstract])) OR (Preinfarction Syndromes, Myocardial[Title/Abstract])) OR (Syndrome, Myocardial Preinfarction[Title/Abstract])) OR (Syndromes, Myocardial Preinfarction[Title/Abstract]) |
|  | #4 | #1 OR #2 OR #3 |
|  | #5 | ((((Danhong injection[Title/Abstract]) OR (Danhong[Title/Abstract])) OR (Dan hong[Title/Abstract])) OR (DHI[Title/Abstract])) OR (danshenhonghua[Title/Abstract]) |
|  | #6 | #4 AND #5 |
| Cochrane  Library | #1 | MeSH descriptor: [Angina, Unstable] explode all trees |
|  | #2 | (Angina, Unstable):ti,ab,kw OR (Anginas, Unstable):ti,ab,kw OR (Unstable Anginas):ti,ab,kw OR (Angina Pectoris, Unstable):ti,ab,kw OR (Angina Pectori, Unstable):ti,ab,kw OR (Unstable Angina Pectori):ti,ab,kw OR (Unstable Angina Pectoris):ti,ab,kw OR (Unstable Angina):ti,ab,kw OR (Angina at Rest):ti,ab,kw OR (Angina, Preinfarction):ti,ab,kw OR (Anginas, Preinfarction):ti,ab,kw OR (Preinfarction Angina):ti,ab,kw OR (Preinfarction Anginas):ti,ab,kw OR (Myocardial Preinfarction Syndrome):ti,ab,kw OR (Myocardial Preinfarction Syndromes):ti,ab,kw OR (Preinfarction Syndrome, Myocardial):ti,ab,kw OR (Preinfarction Syndromes, Myocardial):ti,ab,kw OR (Syndrome, Myocardial Preinfarction):ti,ab,kw OR (Syndromes, Myocardial Preinfarction) |
|  | #3 | #1 OR #2 |
|  | #4 | (Danhong injection):ti,ab,kw OR (Danhong):ti,ab,kw OR (Dan hong):ti,ab,kw OR (DHI):ti,ab,kw OR (danshenhonghua) |
|  | #5 | #3 AND #4 |
| Embase | #1 | "unstable angina pectoris'/exp OR 'unstable angina pectoris |
|  | #2 | 'Angina, Unstable':ab,ti or 'Anginas, Unstable':ab,ti or 'Unstable Anginas':ab,ti or 'Angina Pectoris, Unstable':ab,ti or 'Angina Pectori, Unstable':ab,ti or 'Unstable Angina Pectori':ab,ti or 'Unstable Angina Pectoris':ab,ti or 'Unstable Angina':ab,ti or 'Angina at Rest':ab,ti or 'Angina, Preinfarction':ab,ti or 'Anginas, Preinfarction':ab,ti or 'Preinfarction Angina':ab,ti or 'Preinfarction Anginas':ab,ti or 'Myocardial Preinfarction Syndrome':ab,ti or 'Myocardial Preinfarction Syndromes':ab,ti or 'Preinfarction Syndrome, Myocardial':ab,ti or 'Preinfarction Syndromes, Myocardial':ab,ti or 'Syndrome, Myocardial Preinfarction':ab,ti or 'Syndromes, Myocardial Preinfarction' |
|  | #3 | #1 OR #2 |
|  | #4 | 'Danhong injection':ab,ti or 'Danhong':ab,ti or 'Dan hong':ab,ti or 'DHI':ab,ti or 'danshenhonghua' |
|  | #5 | #3 AND #4 |
| Web of Science |  | TS = (Angina, Unstable OR Anginas, Unstable OR Unstable Anginas OR Angina Pectoris, Unstable OR Angina Pectori, Unstable OR Unstable Angina Pectori OR Unstable Angina Pectoris OR Unstable Angina OR Angina at Rest OR Angina, Preinfarction OR Anginas, Preinfarction OR Preinfarction Angina OR Preinfarction Anginas OR Myocardial Preinfarction Syndrome OR Myocardial Preinfarction Syndromes OR Preinfarction Syndrome, Myocardial OR Preinfarction Syndromes, Myocardial OR Syndrome, Myocardial Preinfarction OR Syndromes, Myocardial Preinfarction) AND TS = (Danhong injection OR Danhong OR Dan hong OR DHI OR danshenhonghua) |
| CNKI |  | SU%=( "不稳定性心绞痛" + "不稳定型心绞痛" + "不稳定心绞痛" + "胸痹") and SU%=("丹红" + "丹红注射液" + "丹参红花" + "丹参红花注射液") |
| Wanfang  Data |  | 主题:( "不稳定性心绞痛" or "不稳定型心绞痛" or "不稳定心绞痛" or "胸痹") and 主题:( "丹红" or "丹红注射液" or "丹参红花" or "丹参红花注射液") |
| VIP |  | (M=(不稳定性心绞痛 + 不稳定型心绞痛 + 不稳定心绞痛 + 胸痹) and (丹红 + 丹红注射液 + 丹参红花 + 丹参红花注射液)) + (R=(不稳定性心绞痛 + 不稳定型心绞痛 + 不稳定心绞痛 + 胸痹) and (丹红 + 丹红注射液 + 丹参红花 + 丹参红花注射液)) |
| CBM | #1 | "心绞痛, 不稳定型"[不加权:扩展] |
|  | #2 | "休息时咽峡炎"[常用字段:智能] OR "心肌梗死前综合征"[常用字段:智能] OR "梗塞前心绞痛"[常用字段:智能] OR "不稳定心绞痛"[常用字段:智能] OR "不稳定型心绞痛"[常用字段:智能] |
|  | #3 | (#2) OR (#1) |
|  | #4 | "丹红"[常用字段:智能] OR "丹红注射液"[常用字段:智能] OR "丹参红花"[常用字段:智能] OR "丹参红花注射液"[常用字段:智能] |
|  | #5 | (#4) OR (#3) |

**Supplementary Table S1.** Search strategies

| **AEs** | **Trial group (n)** | **Control group (n)** |
| --- | --- | --- |
| Gastrointestinal reactions | 17 | 11 |
| Dizziness or headache | 2 | 3 |
| Skin rash | 1 | 0 |
| Light liver function impairment | 1 | 2 |
| Flushed and feverish | 2 | 0 |
| Palpitations | 3 | 0 |
| Gum bleeding | 0 | 1 |
| Unspecified | 12 | 18 |

**Supplementary Table S2.** Summary of AEs in Trial group and Control group

| **Quality assessment** | | | | | | | **No of patients** | | **Effect** | | **Quality** | **Importance** |
| --- | --- | --- | --- | --- | --- | --- | --- | --- | --- | --- | --- | --- |
|  |  |  |  |  |  |  |  |  |  |  |  |  |
| **No. of studies** | **Design** | **Risk of bias** | **Inconsistency** | **Indirectness** | **Imprecision** | **Other considerations** | **DHI** | **non-DHI** | **Relative (95% CI)** | **Absolute** |  |  |
| **hs-CRP** | | | | | | | | | | | | |
| 31 | randomised trials | serious | very serious | no serious indirectness | no serious imprecision | reporting bias | 1597 | 1556 | - | SMD 1.34 lower (1.77 to 0.9 lower) | ⊕OOO VERY LOW | IMPORTANT |
| **TNF-α** | | | | | | | | | | | | |
| 10 | randomised trials | serious | very serious | no serious indirectness | no serious imprecision | none | 491 | 466 | - | SMD 0.84 lower (1.54 to 0.15 lower) | ⊕OOO VERY LOW | IMPORTANT |
| **IL-6** | | | | | | | | | | | | |
| 10 | randomised trials | serious | very serious | no serious indirectness | no serious imprecision | none | 521 | 496 | - | SMD 1.05 lower (1.86 to 0.25 lower) | ⊕OOO VERY LOW | IMPORTANT |
| **NO** | | | | | | | | | | | | |
| 9 | randomised trials | serious | very serious | no serious indirectness | no serious imprecision | none | 447 | 436 | - | SMD 1.51 higher (1.04 to 1.97 higher) | ⊕OOO VERY LOW | IMPORTANT |
| **ET/ET-1** | | | | | | | | | | | | |
| 10 | randomised trials | serious | very serious | no serious indirectness | no serious imprecision | none | 469 | 456 | - | SMD 2.01 lower (2.57 to 1.46 lower) | ⊕OOO VERY LOW | IMPORTANT |
| **Hcy** | | | | | | | | | | | | |
| 7 | randomised trials | serious | no serious inconsistency | no serious indirectness | no serious imprecision | reporting bias | 308 | 295 | - | SMD 0.55 lower (0.71 to 0.39 lower) | ⊕⊕OO LOW | IMPORTANT |
| **AEs** | | | | | | | | | | | | |
| 21 | randomised trials | serious^1^ | no serious inconsistency | no serious indirectness | serious^1^ | none^1^ | 37/975 (3.8%) | 33/966 (3.4%) | OR 1.14 (0.7 to 1.84) | 5 more per 1000 (from 10 fewer to 27 more) | ⊕⊕OO LOW | IMPORTANT |

**Supplementary Table S3.** The summary findings by the grading recommendations assessment, development, and evaluation (GRADE) methods.
